# Labyrinth scale in the differential diagnosis between autism spectrum disorder and language disorder

**DOI:** 10.1590/2317-1782/e20250212en

**Published:** 2026-05-18

**Authors:** Tainá Rossato Benfica, Milena Pondé, Gustavo Marcelino Siquara, Ana Paula Ramos de Souza

**Affiliations:** 1 Universidade Federal de Santa Maria – UFSM - Santa Maria (RS), Brasil.; 2 Escola Bahiana de Medicina - Salvador (BA), Brasil.; 3 Universidade Federal do Rio Grande do Sul – UFRGS - Porto Alegre (RS), Brasil.

**Keywords:** Language, Development, Evaluation, Autism Spectrum Disorder, Developmental Language Disorder

## Abstract

**Purpose:**

To analyze the contribution of the Labyrinth Scale in the differential diagnosis of children with Autism Spectrum Disorder (ASD) and Language Disorder (LD) aged between 2 and 4 years.

**Methods:**

The sample consisted of 38 children diagnosed with LD, 48 with ASD, and 28 with Typical Development (TD). The LD group, evaluated in private clinics and school clinics, consisted of children with language delays confirmed by the Dimensional Inventory of Child Development Assessment (IDADI), as well as speech-language pathology evaluation with instruments such as the Language Development Assessment (LDA), ABFW, among others. They were also evaluated using the Labyrinth Scale. Those responsible signed the Free and Informed Consent Term (FICT). Statistical analysis was performed using JASP software, version 0.15, one-way ANOVA test, and Scheffé post hoc tests.

**Results:**

Differentiated scores were demonstrated between the LD, ASD, and TD groups both in general terms and in the subscales of the Labyrinth Scale.

**Conclusion:**

The Labyrinth Scale allowed differentiating the LD, ASD, and TD groups, showing that the instrument has specificity to perform the differential diagnosis.

## INTRODUCTION

The increase in the prevalence of Autism Spectrum Disorder (ASD) and the previous prevalence of Language Disorder (LD) make both conditions important in terms of public health. ASD diagnoses have increased by 150% since the year 2000, while LD is one of the most frequently found disorders in the child population. It is estimated that ASD affects about 1.5% of the population in certain locations, whereas for LD, studies indicate a variation of approximately 3 to 8% of the affected population^(1)^, as one in every 31 children aged 8 years has a diagnosis of ASD, corresponding to 3.2% of the population.

For the diagnosis of ASD, two broad criteria are defined according to the Diagnostic and Statistical Manual of Mental Disorders - V (DSM-V)^(2)^. The first consists of persistent difficulties in communication and social interaction, and the second comprises restricted and repetitive patterns of behaviors, activities, and interests. The levels vary according to the support needs of the individual and the impairments in adaptive functioning. The gold standard for diagnosis is speech-language pathology assessment, as well as for LD^(2)^. The importance of speech-language pathology assessment following uniform criteria is recognized so that evidence-based practices can be achieved^(3)^ and the lack of professional knowledge regarding diagnostic and intervention processes in speech-language pathology can be overcome^(4)^.

Also, following the DSM-V^2^, within the diagnostic criteria for LD, there are persistent difficulties in the acquisition and use of language across modalities due to deficits in comprehension or production, which include reduced vocabulary, limited sentence structure, and impaired discourse. Moreover, linguistic abilities below what is expected for age result in functional limitations in effective communication, social participation, academic success, or professional performance, individually or in any combination. A recent study also highlights significant deficits in symbolic maturity despite average or above-average intellectual performance, that is, a delay in the mastery of play skills^(5)^.

As observed, the symptoms of both disorders are well defined. However, clinicians may encounter overlap between characteristic symptoms of LD and ASD, which makes establishing the diagnosis challenging. Differentiating ASD and LD seems clear when considering the core symptom of restricted interests and repetitive behaviors present in ASD cases and absent in children with LD, as described in the DSM-V^2^. Nevertheless, when aspects of communication and language, and social interaction are analyzed, there may be overlap in the attribution of similar symptoms, although social skills are distinct in the evaluation of a more experienced clinician.

When subjected to standardized assessments of communicative skills used to assist in establishing diagnoses, children from both studied groups present scores and classifications below expected when compared to typically developing children. However, what is observed is that children with ASD present even greater impairments in the performance of these skills compared to individuals with LD. Furthermore, it is noted that, especially at ages below three years, differential diagnosis is even more complex. In this age group, ASD cases classified as support level one, in which symptomatic manifestations are less evident compared to levels two and three^(6)^, may present a diagnostic interface with cases of LD. Therefore, diagnostic errors are common, in which children with LD characteristics associated with emotional factors receive a medical diagnosis of ASD before speech-language pathology evaluation. The opposite situation also occurs, in which children with ASD support level one may generate doubt regarding the differential diagnosis of LD for the speech-language pathologist. It should be emphasized that both situations were observed in the clinical experiences of the researchers and motivated this study.

Among the challenges involved in establishing a diagnosis that is essentially clinical is deciding which instruments to consider in a multiprofessional assessment. Among these is the Labirinto Scale for ASD diagnosis, which can be applied to children aged two to four years and eleven months by any certified member of the multiprofessional team, with excellent psychometric indices in validation for the Brazilian population^(7)^. This scale assesses the core ASD symptoms described in the DSM-V^2^, organized into the subscales Verbal Communication (VC), Nonverbal Communication (NVC), Social Interaction (SI), and Rigid Behavior and Repetitive Gestures (RBRG). After instrument application, a score is assigned and, according to psychometric data, it is possible to establish the ASD diagnosis. The instrument also allows mapping of associated symptoms (self- and hetero-injury, hyper- and hypoactivity, cognition and impulsivity, disruptive behavior, and obsessive/compulsive behavior) and analyzes the presence or absence of somatic alterations such as epilepsy, respiratory and gastrointestinal diseases, sleep, feeding, and muscle tone.

Based on the assumptions previously presented, this study’s general objective is to analyze the contribution of the Labirinto Scale in the differential diagnosis of children with ASD and LD aged between 2 and 4 years, considering the total score and the scale subdomains (SI, VC, NVC, and RBRG).

## METHOD

This study follows a quantitative and cross-sectional design, constituting an extension of project number 3.119.402, previously approved by the Research Ethics Committee (REC) of the Escola Bahiana de Medicina. The study procedures were conducted in accordance with the guidelines and regulatory standards for research involving human subjects, as established by the Brazilian National Health Council (CNS) in resolutions 466/12 and 510/16. The research participants and their guardians only took part in the study after reading and signing the Free and Informed Consent Term (FICT), upon full knowledge of the research objectives and procedures.

Part of the data consisted of secondary data from the Typical Development (TD) and ASD groups, derived from the original study. The administrators of the sites where data were collected consented to the procedures, granting authorization for the research, reviewed by the REC of the institution where the study was registered. Data from the LD group were collected at a different time to allow comparison among the three groups.

### Participants

The sample consisted of 38 children with LD, 48 children with ASD, and 28 children with TD. Data from children with ASD and TD were obtained from a database provided to this study by the laboratory responsible for the original Labirinto Scale research^(7)^. A sample of 45 children with LD had initially been proposed based on sample size calculation using the existing database. However, due to the ongoing COVID-19 pandemic, a convenience sample composed of 38 children assessed with the Labirinto Scale by the researchers was adopted. The terminology LD was used instead of Developmental Language Disorder in order to follow DSM-V^2^ criteria.

In the LD group, children aged between two and four years and eleven months were included, respecting the age range defined for application of the scale. Participants recruited from private clinics were required to have a previous diagnosis of LD performed by a speech-language pathologist with clinical experience and specialization in language (all were master’s students in language disorders). Children from teaching clinics of public institutions were required to be on the waiting list and present a primary complaint of language delay, or to be receiving care from speech-language pathologists specialized in language. They were called and assessed by the research team under supervision of the main advisor of this study, who has more than 30 years of experience with LD. Additionally, some children already receiving treatment in teaching clinics with a previous diagnosis of LD were invited to participate and were assessed by this study’s researcher and/or advisor. For confirmation of the LD diagnosis, it was verified that all responsible professionals considered DSM-V^2^ diagnostic criteria, regardless of the previous assessments applied to establish this diagnostic hypothesis.

An additional inclusion criterion required that children present evidence of delay or significant delay in receptive and/or expressive communication and language domains on the IDADI^(8)^. The IDADI evaluates cognition, socioemotional development, receptive and expressive language and communication, gross motor skills, fine motor skills, and adaptive behavior.

Exclusion criteria included children with malformations, syndromes, hearing and/or visual and/or motor impairment, children undergoing bilingual language acquisition processes, those with cognitive deficits suggesting significant intellectual disability, or with an ASD diagnosis established by a physician and/or by the standard score of the Labirinto Scale^(7)^.

The ASD group, whose data were provided by the scale’s research laboratory, had as inclusion criteria a previous medical diagnosis of ASD and scores compatible with the diagnosis according to CARS^(9)^ and the Labirinto Scale^(7)^. The TD group, also collected by the same laboratory, had as inclusion criteria the absence of delays, deficits, or neurodevelopmental disorders and scores below the cutoff point for ASD diagnosis according to CARS^(9)^. To minimize potential bias resulting from the LD group being collected at a different time and setting, doubtful cases in the differential diagnosis were reviewed by the Labirinto group in sessions conducted by Dr. Milena Pondé and Dr. Gustavo Siquara, authors of the scale. During this process, the group was blinded to whether the case corresponded to LD or ASD and sought to establish the differential diagnosis. The speech-language pathologist researchers were also certified in the application of the Labirinto Scale by both authors and participated in weekly diagnostic follow-up sessions during one year of training.

### Instruments and data collection procedures

In the first stage, contact was made with the teaching clinics of two public Speech-Language Pathology programs and with speech-language pathologists from private clinics (six in total) to locate participants with LD characteristics or complaints of delayed language acquisition. Subsequently, contact was made with the caregivers in person or via WhatsApp, inviting them to participate in the study. An in-person evaluation was scheduled for those who agreed to volunteer.

In addition to scheduling, at this stage the Labirinto Scale anamnesis was sent to caregivers via Google Forms. The Dimensional Inventory of Child Development Assessment (*Inventário Dimensional de Avaliação do Desenvolvimento Infantil* - IDADI)^(8)^ was also sent online through the test publisher for caregivers to complete. It should be noted that online administration was performed for participants with digital access. Those who had difficulties or limited access completed the protocols in printed form during the in-person session.

The next stage consisted of an in-person appointment with the child and caregivers for administration of the Labirinto Scale. The first step with the caregivers was the in-person reading of the FICT and obtaining their signature, since they had already agreed to participate during the virtual contact. At this time, caregivers who had not completed the Labirinto Scale anamnesis and the IDADI^(10)^ online filled them out in printed form, and parents were also interviewed to complement anamnesis information and share their perceptions about the child.

Subsequently, the Labirinto Scale^(7)^ was administered through playful procedures in person with the children. The application took place in an individual clinical room, ventilated and well-lit, following the current sanitary requirements, as the COVID-19 pandemic was still ongoing at the beginning of data collection. Assessments were conducted by this study’s researcher or advisor, both certified to perform the evaluation. The application occurred in the presence of a caregiver and lasted approximately 40 to 60 minutes. The materials required for the Labirinto Scale administration were used.

In phase one: animal shape sorter board, two rattles, stacking box tower, ball, toy car, key house, toy phone, and bowling set. In phase two: doll, toy kitchen accessories – miniature stove, pans, baby bottle and toy food, miniature toothbrush, hairbrush, and bed/crib. In some cases, soap bubbles or a balloon were also used, as described in the application guidelines^(7)^.

The evaluations were video recorded using a mobile phone mounted on a tripod stand in the room. Recordings were organized and stored using Google Drive. During the first five minutes of application, the child’s free exploration of toys available in the environment was observed. Afterwards, the planned activities for assessment of the test instructions were initiated.

The Labirinto Scale^(7)^ is a validated instrument for the Brazilian population designed to assess and characterize the core ASD symptoms described in DSM-V^2^ in children aged two to four years and eleven months. The tool is subdivided into Core Symptoms, Associated Symptoms, and Associated Somatic Alterations. It consists of a structured interview and assessment of core symptoms, including the domains Social Interaction (SI), Verbal Communication (VC), Nonverbal Communication (NVC), and Rigid Behavior and Repetitive Gestures (RBRG), as well as mapping of symptoms commonly associated with ASD and associated somatic alterations. Based on systematic and methodical analysis of the four axes that constitute the Core Symptoms, using a Likert scale with scores ranging from zero to five points that characterize frequency and intensity of symptoms, the cutoff point for ASD diagnosis is defined in the total score and in each subscale^(7)^.

For analysis of core symptoms, the Rigid Behavior and Repetitive Gestures (RBRG) subscale evaluates play skill level (simple, combined, symbolic, stereotyped and repetitive), rigidity, repetitive movements, sensory-seeking movements and restricted interests; Nonverbal Communication (NVC) includes response to name, eye contact, initiation and response to joint attention and communicative gestures; Verbal Communication (VC) includes expressive verbal language, linguistic repertoire based on expected age parameters such as word use and narrative, stereotyped language and communicative reciprocity related to participation in dialogue by addressing or responding to the interlocutor; Social Interaction (SI) includes response to approach, seeking the examiner or caregiver during play and social smile.

The first procedure in the evaluation process is the anamnesis. The second stage consists of administering the scale through playful activities. This part is carried out in the presence of the child, the professional, and the caregiver. It is recommended that it take place in an individual room and is estimated to last between 40 and 60 minutes, with video recording. Assessment procedures include observation of spontaneous play initiated by the child during the first five minutes of application. Afterwards, the evaluator conducts specific joint activities in a naturalistic play context, using the materials described in the manual for phases one and two.

The Labirinto Scale also analyzes associated symptoms, including items related to Cognition, Disruptive Behavior, Impulsivity, Hyperactivity, Hypoactivity, Hetero- and Self-injurious Behavior, and Obsessive/Compulsive Behavior. Within Somatic Alterations, the following items are included: Sphincter Control Alterations, Sleep, Gastrointestinal and Respiratory Problems, Tone, Syndromes, Neurological Alterations or Dysmorphisms, Pre- or Perinatal Episodes, and Epilepsy.

At the end of administration, the recording must be analyzed by a professional certified to apply the Labirinto Scale. Scores are assigned on the response sheet, and total and subscale scores are calculated. According to psychometric criteria, the cutoff point for ASD must be equal to or greater than 12 points in the total score, equal to or greater than three in the SI subscale, equal to or greater than four in the VC and RBRG subscales, and equal to or greater than two in the NVC subscale.

For diagnosis of the LD group, in addition to the application of the IDADI^(8)^ by the researchers and the Labirinto Scale^(7)^, other protocol-based assessments were used to assist diagnosis, selected by the speech-language pathologists who evaluated the children before the research was conducted. Moreover, all reported or used DSM-V^2^ criteria to identify the clinical manifestations of LD symptoms. Professionals used tests they considered relevant to assess language acquisition, such as the Language Development Assessment (LDA)^(11)^ or more specific assessments of vocabulary, fluency, pragmatics, phonology (ABFW)^(10)^, and praxis^(12)^.

The assessments of the 28 children in the TD group and the 48 participants in the ASD group were conducted in a psychology clinic, after reading and signing the FICT by caregivers. Psychology trainees and psychiatry residents from Escola Bahiana de Medicina administered the standard Labirinto Scale^(7)^ and CARS^(9)^ procedures to children in both groups, under the supervision of a psychiatrist and a psychologist. Afterwards, the same team scored the scales and performed the clinical diagnosis according to DSM-V^2^ criteria.

The LD group was composed in two ways: referrals from speech-language pathologists in private clinics and selection from the waiting list of teaching clinics. Some children from private clinics had neuropediatric evaluations with an LD diagnosis in addition to speech-language pathology assessment. Thus, an effort was made to use a procedure similar to that used in the ASD group data collection, which included expert clinical evaluation and a specific test, in the case of children with ASD CARS and, in the case of speech therapists, language tests. Children from teaching clinics were selected from a waiting list of children with complaints of delayed language acquisition, and the Labirinto Scale and IDADI were applied to identify potential ASD or LD cases. Language tests and clinical observation by researchers experienced in language were also used. For every ten children with this complaint, four were ASD cases, and six were LD. The Labirinto Scale proved to be fundamental in distinguishing doubtful cases, as previously mentioned, by the Labirinto research group.

### Data analysis

The audiovisual material recorded during the administration of the Labirinto Scale was analyzed by this study’s researcher, who assigned the Labirinto Scale scores for each evaluated case. The study advisor reviewed the established scores. In cases where there was doubt regarding the results, especially when the interface between LD and ASD diagnosis was present, and in cases with evident clinical signs of ASD, the co-advisor verified the tabulations and scoring. If doubts persisted, the case was taken to a study group discussion within the research laboratory. There were no disagreements greater than 5% in the assigned scores, nor in the diagnoses. For the final score decision, the assignments of the advisors of this study prevailed.

The IDADI^(12)^ consists of a comprehensive assessment of child development involving seven domains: cognitive, socioemotional, receptive and expressive communication and language, gross and fine motor skills, and adaptive behavior. The inventory includes 524 items that describe behaviors and skills expected for each age range between four and 72 months of life, respecting the child neurodevelopmental milestones. The protocol is validated for the Brazilian population and allows collection of developmental information through parental reports.

The questionnaire may be completed by parents or caregivers, either in a self-administered format or as an interview conducted by the professional/researcher. Scoring is performed using a Likert scale, in which the response “yes” corresponds to two points when the child masters the behavior described in the item; “sometimes”, when the child performs with difficulty or only occasionally, corresponding to one point; and “no”, when the child does not perform or has never been observed performing the activity, representing zero points. At the end, the raw score for each domain is calculated. Subsequently, standardized and developmental scores can be consulted, which in this study were obtained through the publisher’s online system and sent by email to the study advisor and to the parents and speech-language pathologists responsible for the cases.

Among the analysis options are children who present development far above expected, above expected, typical, at risk for delay, with delay, or with significant delay. In this study, its use aimed to confirm the LD diagnosis assigned by the speech-language pathologists who referred participants to the research. Inclusion criteria required the presence of delay or significant delay in expressive and/or receptive communication and language. Delays or risk for delay could also be present in other evaluated domains, provided they did not characterize cases of intellectual or motor disability, among others, as analyzed by specialists. In cases of doubt, participants were excluded or referred for further assessments.

Afterwards, the revised response data were entered into an Excel spreadsheet containing Labirinto Scale information regarding total score and subscales, considering the three studied groups (TD, ASD and LD).

The analysis of relationships between the collected variables was performed using the JASP software, version 0.15. Descriptive and inferential statistical measures were used. To compare scale scores among the TD, LD, and ASD groups, a one-way ANOVA test was applied. The Scheffé post hoc test was also used to evaluate overall confidence levels when group sample sizes differ, as in this study. It should be noted that the analysis materials and codes are available upon request by email to the corresponding author.

## RESULTS

Table 1 presents the means and standard deviations for the TD, ASD, and LD groups regarding scores in each subscale and in the total score of the Labirinto Scale.

**Table 1:** Group scores on the Labirinto Scale

**Groups**	TD	ASD	LD	ANOVA
Labirinto Subscales	N=28	N=48	N=38	p-value
	M(SD)	M(SD)	M(SD)	Three-group comparison
Social Interaction	0.5 (1.2)	6.9 (2.9)	1.4 (1.4)	<0.001
Verbal Communication	1.3 (2.1)	9.5 (3.2)	6.8 (2.1)	<0.001
Nonverbal Communication	0.7 (2.2)	13.4 (5.7)	3.3 (2.4)	<0.001
Rigid Behaviors and Repetitive Gestures	0.8 (1.1)	6.5 (2.6)	2.4 (1.3)	<0.001
Total Labirinto Score	3.5 (5.2)	37.1 (12.9)	14.1 (4.9)	<0.001

**Caption:** TD = typical development; ASD = autism spectrum disorder; LD = language disorder; N = number; M = mean; SD = standard deviation

In the comparison among groups of children according to diagnosis, using the one-way ANOVA test, a significant difference was observed among the TD, LD and ASD groups for each subscale: Social Interaction (p<0.001), Verbal Communication (p<0.001), Nonverbal Communication (p<0.001), Rigid Behavior and Repetitive Gestures (p<0.001), and Total Labirinto Scale Score (p<0.001). In addition, a progressive increase in scores can be observed, differentiating the TD, LD, and ASD groups across all subscales and in the total score.

**Figure 1:**
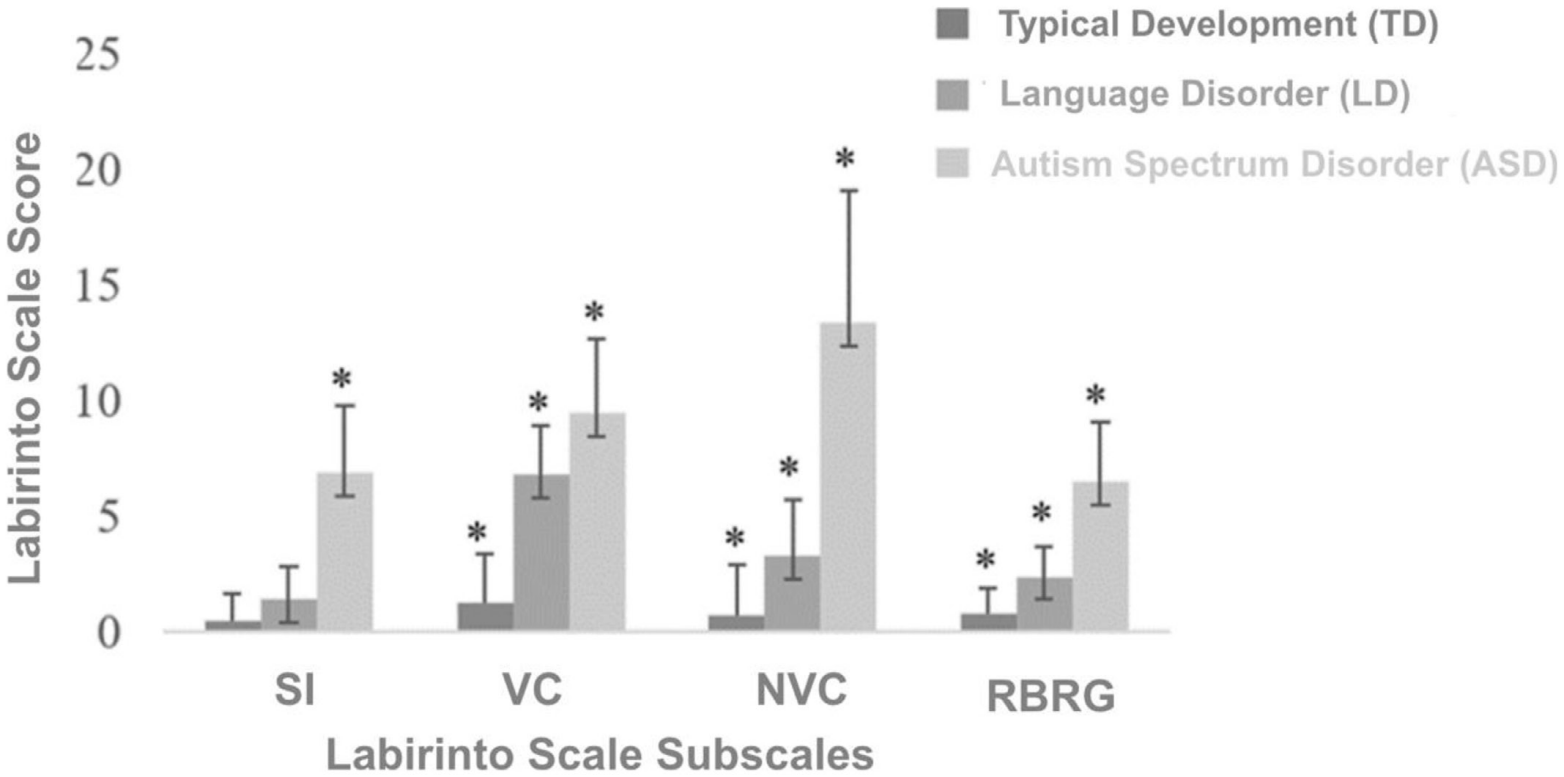
Post hoc comparison among TD, LD, and ASD groups

Figure 1 presents the comparison of scores according to each Labirinto Scale subscale for the TD, LD, and ASD groups.

**Caption:** SI = Social Interaction; VC = Verbal Communication; NVC = Nonverbal Communication; RBRG = Rigid Behavior and Repetitive Gestures. Source: author’s production

According to the analysis of Figure 1, pairwise comparisons analyzed using the Scheffé-corrected post hoc test indicated significant differences among groups in the subscales. In Social Interaction, significant differences were found between the TD and ASD groups (p<0.001) and between the LD and ASD groups (p<0.001). However, no significant differences were found between the TD and LD groups (p=0.310).

Regarding comparison in the Verbal Communication subscale, differences were found among all three compared groups (TD X LD; TD X ASD; LD X ASD) with p<0.001. In the Nonverbal Communication subscale, differences were observed among the compared groups (TD X ASD; LD X ASD) with p<0.001 and TD X LD with p=0.04. Regarding performance comparison in the Rigid Behavior and Repetitive Gestures subscale, differences were found among all compared groups (TD X LD; TD X ASD; LD X ASD) with p<0.001.

Furthermore, analysis of Figure 1 shows a progressive increase in scores in each subscale, following the order TD, LD and ASD groups. In all subscales, the ASD group presented higher scores. Although all subscale scores showed statistically significant differences compared to the ASD group, a greater difference between the LD and ASD groups was observed in the Nonverbal Communication subscale and a smaller difference in Verbal Communication item scores.

**Figure 2:**
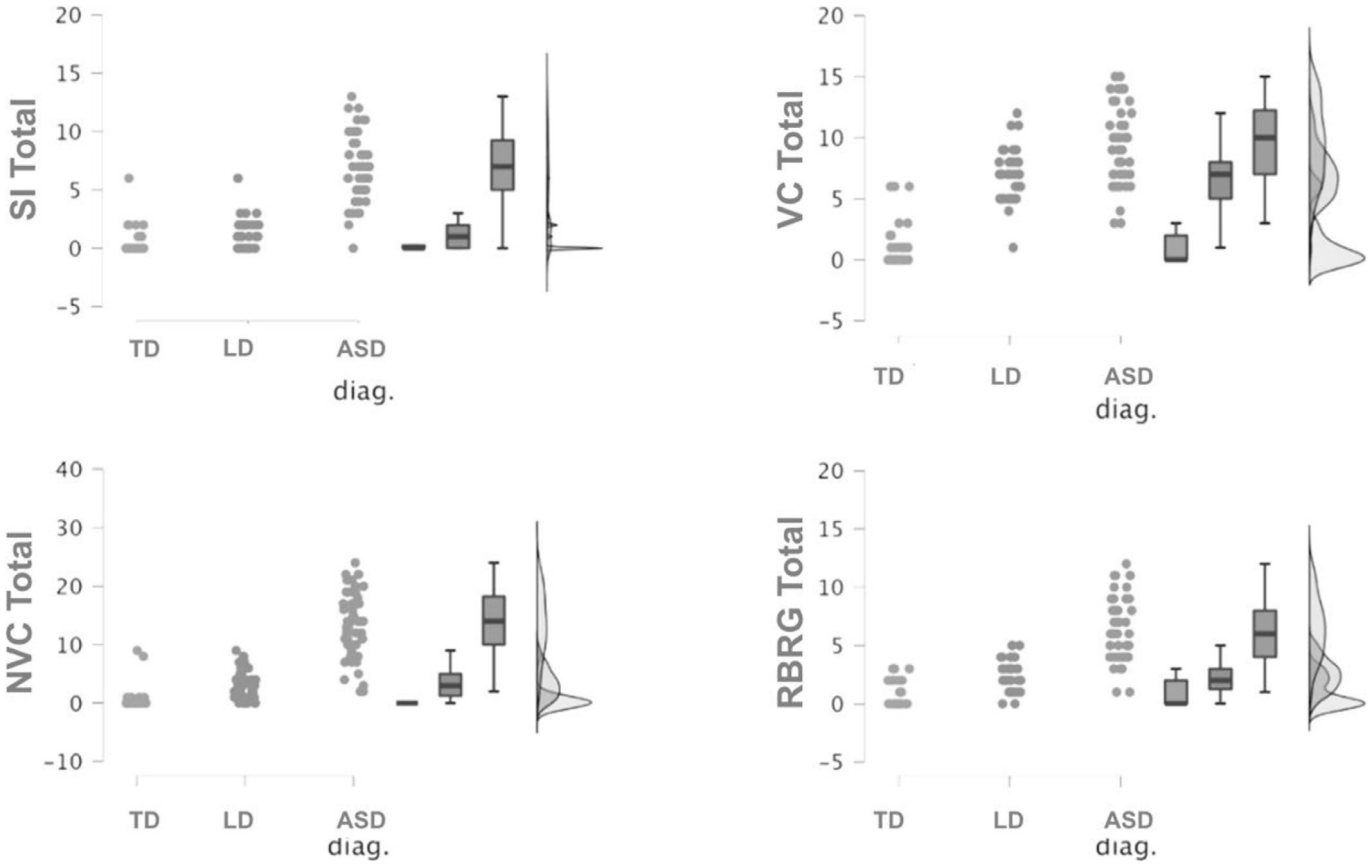
Distribution graphs of TD, LD, and ASD group scores on the Labirinto Scale subscales

The differences found in the subscales assessed by the Labyrinth Scale between the DT, TL, and TEA groups are also shown in Figure 2.

**Caption:** SI = Social Interaction; VC = Verbal Communication; NVC = Nonverbal Communication; RBRG = Rigid Behavior and Repetitive Gestures; TD = Typical Development; LD = Language Disorder; ASD = Autism Spectrum Disorder. Source: author’s production

In Figure 2, it is observed that the score in each evaluation subscale clearly differentiates children with TD and LD from children with ASD. Regarding the core symptom subscales related to Verbal Communication, Nonverbal Communication, and Rigid Behavior and Repetitive Gestures, a statistical difference was found between children with TD and LD. Only the Social Interaction symptom did not allow statistical differentiation between the TD and LD groups.

It is also worth noting that the subscales relate to rigid behaviors and repetitive gestures in children with LD. It was observed that the highest scores were related to the subitem concerning play skills below expectations for the age group, and less to the presence of mannerisms or repetitive gestures.

## DISCUSSION

The Labirinto Scale^(7)^ is an accessible instrument previously validated for the Brazilian context to characterize and assist clinicians in diagnosing ASD in children aged two to four years and eleven months in the Brazilian population. This study’s results indicate that the scale may assist in the differential diagnosis between children with ASD and LD, considering that the scores of all subscales that assess Core Symptoms showed statistically significant differences between the LD and ASD groups. The Social Interaction, Nonverbal Communication, Verbal Communication, and Rigid Behavior and Repetitive Gestures subscales also differentiated typically developing children from those with LD in three of the four evaluated dimensions. The LD and ASD groups presented similar clinical symptoms; however, higher scores were observed in the ASD group compared to the LD group. Thus, the Labirinto Scale may be an instrument capable of discriminating cases in which diagnostic doubts may arise, as highlighted by Markiewicz and Pachalska^(6)^, who emphasize the inherent difficulties in the differential diagnosis between ASD and LD in children under three years of age due to the shared interface of language impairments in both conditions.

The initial complaint of language delay is commonly associated with the absence or presence of speech, an aspect that became particularly concerning during the COVID-19 pandemic^(13)^, when this research was conducted. This factor led caregivers to seek services for both ASD and LD cases. It is important to highlight that although parents tend to notice speech alterations more readily, communicative deficits are not restricted to oral language but also involve gestural communication. This is clearly demonstrated in the analysis of Figures 1 and 2. In the Verbal and Nonverbal Communication graphs of Figure 2, this becomes very evident. Not only did the ASD and LD groups present deficits in the Verbal Communication subscale, but delays were also evident in the Nonverbal Communication subscale in both groups, more prominently in the ASD group. This demonstrates how the interface between oral and gestural communication becomes substantial when language is understood from a multimodal perspective, in which a cognitive-linguistic functional matrix is constructed, and gesture is integrated with vocal production^(14)^.

Therefore, when there is evident delay in verbal communication, nonverbal communication skills must also be observed. In this study, the Labirinto Scale^(7)^ proved capable of identifying delay in both subscales, with considerably higher scores in ASD compared to the LD group.

These factors have been demonstrated in other studies, which indicate that ASD involves atypical patterns in the use of gestures with communicative function. In addition to producing fewer gestures, children with ASD show deficits particularly in gestures related to joint attention skills and in integrating gaze and gesture for communication, especially from 12–13 months of age^(15)^. These findings highlight the importance of considering nonverbal communication scores in the differential diagnosis among ASD, LD, and TD.

In the Social Interaction subscale, the LD group did not show significant differences compared to the TD group, as represented in Table and Figure 1. This aspect may indicate that children with LD do not necessarily present difficulties in social interactions, given their intellectual and socioemotional conditions, as suggested by classical literature that once described these disorders as specific to language^(14)^.

In this same subscale, however, regarding the distinction between ASD and LD groups, score differences are clear. The ASD group scored significantly higher than the LD group. It is worth mentioning the study by Simms and Jin^(15)^, which analyzed differences between symptoms in children with ASD and LD according to DSM^(2)^ and indicated that children with LD present greater social interest, more consolidated joint attention skills, and greater affective reciprocity. The same study highlighted that deficits in social interaction may occur, but as a consequence of basic language difficulties.

In the last analyzed subscale, as shown in Figures 1 and 2, the Rigid Behavior and Repetitive Gestures subscale followed the same pattern observed in the other subscales. The ASD group presented statistically higher scores compared to the LD and TD groups. This result is consistent with behaviors already described in the literature regarding ASD symptomatology. Children with ASD demonstrate more restricted interests and hyperfocus on specific topics or objects that limit their interest in others, inflexibility and rigidity with routines, atypical object manipulation, and vocal or motor stereotypies^(2,16)^.

Although they obtained lower scores than the ASD group in the Rigid Behavior and Repetitive Gestures subscale, it is noteworthy that LD cases scored significantly higher than TD cases. Among the four items composing this domain, the LD group showed higher scores mainly in repetitive/stereotyped movements and play/symbolization.

In this context, cases of motor stereotypies in children with LD, commonly considered a warning sign for ASD, have already been described in a study that identified motor stereotypies similar to those characteristic of ASD in two children with LD. Another study analyzing 277 children with different diagnoses, including ASD, found that 18.3% of children with LD presented some type of stereotypy^(17)^.

Another factor contributing to the increased scores of the LD group, differentiating it from the TD group in the Rigid Behavior and Repetitive Gestures subscale, was the play skills item. Both language and symbolic play require representational capacity, abstraction, and imagination^(18,19)^. Children who achieve more complex levels of play also demonstrate better performance in cognitive and communicative assessments^(18,19)^.

In children with LD, poorer play skills are observed. The profile of characteristics found in children with LD indicates fewer variations in play, fewer initiations of routines, a smaller play repertoire, and especially fewer symbolic behaviors at the pretend-play level^(19-23)^. This was confirmed in this study since, although the ASD group presented the highest scores in this subscale item, indicating a more restricted play pattern with fewer functional behaviors, the LD group, to a lesser extent, also showed difficulties in demonstrating creative and flexible play skills, especially at symbolic levels, such as attributing life to a doll, using an object as if it were something else and imagining the presence of an object without its concrete presence^(18)^.

Among the strengths of using the Labirinto Scale are the joint training of the multidisciplinary team and the comprehensive developmental framework that underlies it, since there are developmental parameters that serve as references and create a baseline for observation based on video-recorded tasks, minimizing subjective interpretation. Another strength is its financial accessibility to clinical professionals, either through online training that can be accessed from any region of Brazil or through the affordable acquisition of toys and materials required for its administration. These strengths were reflected in this research. Furthermore, the fact that data collection for the LD group occurred in different cities within the state and included cases attended in both teaching clinics and private clinics represents another strength due to the diversity of economic and cultural contexts represented.

Regarding this study’s limitations, it should be noted that joint and blinded data collection for both groups, ASD and LD, was not possible. Since the LD study was conducted shortly after the scale validation, it is believed that future studies may minimize this limitation.

## CONCLUSION

The Labirinto Scale proved effective in differentiating between the ASD group and the LD group, as well as between children with ASD and those with TD. All subscales also differentiated the three groups, except for the Social Interaction subscale, which did not show a significant distinction between the LD and TD groups.

The study suggests that, for more robust confirmation, the scale should be investigated with larger samples and in clinical situations in which the diagnosis is unknown, to verify its effectiveness in differential diagnosis.
