# Supplementary material for: Labyrinth scale in the differential diagnosis between autism spectrum disorder and language disorder
Source: Codas. 2026 May 18;38(3):e20250212. doi: 10.1590/2317-1782/e20250212en (PMC13193194; doi:10.1590/2317-1782/e20250212en)
Supplement: Translation

# Translation: Escala labirinto no diagnóstico diferencial entre o transtorno do espectro autista e o transtorno de linguagem

**DOI:** 10.1590/2317-1782/e20250212pt

**Authors:** Tainá Rossato Benfica, Milena Pondé, Gustavo Marcelino Siquara, Ana Paula Ramos de Souza

**Affiliations:** 1 Universidade Federal de Santa Maria – UFSM - Santa Maria (RS), Brasil.; 2 Escola Bahiana de Medicina - Salvador (BA), Brasil.; 3 Universidade Federal do Rio Grande do Sul – UFRGS - Porto Alegre (RS), Brasil.

**Keywords:** Linguagem, Desenvolvimento, Avaliação, Transtorno do Espectro Autístico, Transtorno do Desenvolvimento da Linguagem

## Abstract

**Objetivo:**

Analisar a contribuição da Escala Labirinto na realização do diagnóstico diferencial de crianças com Transtorno do Espectro Autista (TEA) e Transtorno de Linguagem (TL) com idade entre 2 e 4 anos.

**Método:**

A amostra foi composta por 38 crianças com diagnóstico de TL, 48 com TEA e 28 com desenvolvimento típico (DT). O grupo TL, avaliado em clínicas particulares e clínicas-escola, constou de crianças com atraso de linguagem comprovado pelo Inventário Dimensional de Avaliação do Desenvolvimento Infantil (IDADI), bem como avaliação fonoaudiológica com instrumentos como Avaliação do desenvolvimento da linguagem (ADL), ABFW, entre outros. Também foram avaliadas pela Escala Labirinto. Os responsáveis assinaram o termo de consentimento livre e esclarecido. A análise estatística foi realizada por meio do software JASP, versão 0.15., teste ANOVA one-way e teste Post Hoc Scheffe.

**Resultados:**

Demonstraram pontuações diferenciadas entre os grupos TL, TEA e DT tanto em termos gerais quanto nas subescalas da Escala Labirinto.

**Conclusão:**

A Escala Labirinto permitiu diferenciar os grupos TL, TEA e DT, evidenciando que o instrumento apresenta especificidade para realizar o diagnóstico diferencial.

## INTRODUÇÃO

O aumento na prevalência do Transtorno do Espectro Autista (TEA) e a prevalência prévia de Transtorno de Linguagem (TL) tornam ambos distúrbios importantes em termos de saúde pública. Os diagnósticos de TEA sofreram um aumento de 150% desde o ano 2000, enquanto o TL é um dos distúrbios mais encontrados na população infantil. Estima-se que o TEA atinge cerca de 1,5% da população em determinados locais, enquanto para o TL, estudos apontam uma variação de cerca de 3 a 8% da população acometida^(1)^, pois uma a cada 31 crianças de 8 anos possui diagnóstico de TEA, correspondendo a 3,2% da população.

Para o diagnóstico de TEA são definidos dois critérios amplos, conforme Manual Diagnóstico e Estatístico de Transtornos Mentais - V (DSM-V)^(2)^. O primeiro consiste em dificuldades persistentes na comunicação e interação social e o segundo compreende a padrões restritos e repetitivos de comportamentos, atividades e interesses. Os níveis variam de acordo com a necessidade de suporte que o sujeito necessita e com os prejuízos no funcionamento adaptativo. O padrão-ouro para diagnóstico é avaliação fonoaudiológica, assim como para o TL ^2^. Sabe-se da importância de a avaliação fonoaudiológica seguir critérios uniformes para que sejam possíveis práticas baseadas em evidências^(3)^ e o déficit no conhecimento dos profissionais referente aos processos de diagnóstico e intervenção fonoaudiológica sejam superados^(4)^.

Seguindo também o DSM-V^2^, dentro dos critérios diagnósticos do TL, apresentam-se dificuldades persistentes na aquisição e no uso da linguagem em suas diversas modalidades devido a déficits na compreensão ou na produção, que incluem vocabulário reduzido, estrutura limitada de frases e prejuízo no discurso. Além disso, capacidades linguísticas abaixo do esperado para a idade, resultando em limitações funcionais na comunicação efetiva, na participação social, no sucesso acadêmico ou no desempenho profissional, individualmente ou em qualquer combinação. Estudo recente evidencia também déficits importantes na maturidade simbólica, apesar do desempenho médio ou superior em termos intelectuais, ou seja, um atraso no domínio dos jogos^(5)^.

Como se observa, os sintomas de ambos os transtornos são bem definidos. No entanto, os clínicos podem encontrar sobreposição de sintomas característicos de TL e TEA, o que torna o estabelecimento do diagnóstico desafiador. Diferenciar TEA e TL parece claro quando considerado o sintoma central de interesses restritos e comportamentos repetitivos presentes nos casos de TEA e ausente nas crianças com TL, conforme descrito no DSM-V^2^. No entanto, quando são analisados aspectos da comunicação e linguagem, e interação social, pode haver interface na atribuição de sintomas similares, embora as habilidades sociais sejam distintas na avaliação do clínico mais experiente.

Quando submetidas a avaliações padronizadas de habilidades comunicativas utilizadas para auxiliar no estabelecimento dos diagnósticos, crianças de ambos os grupos estudados apresentam pontuações e classificações abaixo do esperado se comparadas às crianças em desenvolvimento típico. Entretanto, o que se observa é que as crianças com TEA apresentam ainda mais prejuízos no desempenho dessas habilidades em relação aos sujeitos com TL. Ainda, observa-se que, sobretudo em idades inferiores a três anos, o diagnóstico diferencial é ainda mais complexo. Observa-se, nessa faixa etária, que casos de TEA classificados como nível de suporte um, nos quais as manifestações sintomáticas são menos evidentes em comparação aos níveis dois e três^(6)^, pode haver uma interface diagnóstica com casos de TL. Por isso, é comum observar erros diagnósticos, nos quais crianças com características de TL associadas a fatores emocionais receberem o diagnóstico médico de TEA, antes da avaliação fonoaudiológica. A situação contrária também ocorre em que essas crianças com TEA nível de suporte um podem gerar dúvida quanto ao diagnóstico diferencial de TL no profissional fonoaudiólogo. Ressalta-se que ambas as situações foram observadas nas vivências clínicas das pesquisadoras e motivaram este estudo.

Dentre os impasses que envolvem o estabelecimento de um diagnóstico que é essencialmente clínico, está quais instrumentos considerar em uma avaliação multiprofissional. Dentre estes, tem-se a Escala Labirinto para diagnóstico de TEA, que pode ser aplicada na faixa etária de dois a quatro anos e 11 meses por qualquer membro certificado da equipe multiprofissional, com índices psicométricos excelentes na validação em população brasileira^(7)^. Esta escala avalia os sintomas centrais do TEA descritos no DSM-V^2^, que estão organizados nas subescalas de Comunicação Verbal (CV), Comunicação Não Verbal (CNV), Interação Social (IS) e Comportamento Rígido e Gestos Repetitivos (CRGR). Após a aplicação do instrumento, é atribuída a pontuação e, conforme dados psicométricos, é possível estabelecer o diagnóstico de TEA. O instrumento permite ainda o mapeamento de sintomas associados (auto e heterolesão, hiper e hipoatividades, cognição e impulsividade, comportamento disruptivo e comportamento obsessivo/compulsivo) e analisa a presença ou não de alterações somáticas como: epilepsia, doenças respiratórias e do trato gastrointestinal, sono, alimentação e tônus.

A partir dos pressupostos elencados anteriormente, o objetivo geral deste estudo é analisar a contribuição da Escala Labirinto na realização de diagnóstico diferencial de crianças com TEA e TL com idades entre 2 e 4 anos, considerando a pontuação geral e os subdomínios da escala (IS, CV, CNV E CRGR).

## MÉTODO

Essa pesquisa segue um delineamento quantitativo e transversal, constituindo-se numa extensão do projeto de número 3.119.402, que apresenta aprovação prévia no Comitê de Ética em Pesquisa da Escola Bahiana de Medicina. As etapas de estudo foram realizadas conforme as diretrizes e normas regulamentadoras de pesquisas envolvendo seres humanos, no que determina o Conselho Nacional de Saúde em suas resoluções 466/12 e 510/16. Os sujeitos da pesquisa e seus responsáveis somente participaram do estudo mediante leitura e assinatura do Termo de Consentimento Livre e Esclarecido (TCLE), após pleno conhecimento dos objetivos e procedimentos da pesquisa.

Utilizou-se uma parte de dados secundários, dos grupos em desenvolvimento típico (DT) e com TEA, provenientes da pesquisa original. Os responsáveis pelos locais onde foram coletados os dados consentiram os procedimentos, oferecendo autorização para realização da pesquisa, revisada no CEP da instituição de registro da pesquisa. Os dados do grupo com TL foram coletados em outro momento para possibilitar a comparação dos três grupos.

### Participantes

A amostra constituiu-se de 38 crianças com TL, 48 crianças com TEA e 28 crianças com DT. Os dados das crianças com TEA e DT fazem parte de um banco de dados cedido a este estudo pelo laboratório da pesquisa original com a Escala Labirinto^(7)^. Havia uma proposta de 45 crianças com TL considerando o cálculo amostral a partir do banco de dados existente. No entanto, considerando a pandemia da COVID 19 em curso, assumiu-se uma amostra de conveniência composta por 38 crianças avaliadas com a Escala Labirinto pelas pesquisadoras. Considerou-se a nomenclatura TL ao invés de Transtorno do Desenvolvimento da Linguagem, para seguir os critérios do DSM-V^2^.

No grupo com TL foram incluídas crianças na faixa etária de dois a quatro anos e 11 meses, respeitando a faixa etária definida para aplicação da escala. Os sujeitos provenientes das clínicas particulares deveriam apresentar diagnóstico prévio de TL realizado por fonoaudiólogo com experiência clínica e especializado em linguagem (todos eram mestrandos na área dos distúrbios de linguagem). As crianças das clínicas-escola de instituições públicas deveriam estar na fila de espera e apresentar queixa principal de atraso de linguagem, ou em consultório de fonoaudiólogas especialistas em linguagem. Elas foram chamadas e avaliadas pela equipe de pesquisa, com supervisão da orientadora principal deste trabalho que apresenta mais de 30 anos de experiência com TL. Também, algumas crianças que já estavam em atendimento nas clínicas-escola com diagnóstico prévio de TL foram chamadas para a pesquisa e foram avaliadas pela pesquisadora e/ou orientadora desse estudo. Para conclusão do diagnóstico de TL verificou-se que todos os profissionais responsáveis consideravam os critérios diagnósticos do DSM-V^2^, independentemente das avaliações prévias que tais profissionais tenham aplicado para fazer essa hipótese diagnóstica.

Utilizou-se ainda, como critério adicional de inclusão na amostra, as crianças apresentarem evidências de atraso ou atraso significativo nas dimensões de comunicação e linguagem receptiva e/ou expressiva no IDADI^(8)^. O IDADI avalia as dimensões de cognição, socioemocional, linguagem e comunicação receptiva e expressiva, motricidade ampla, motricidade fina e comportamento adaptativo.

Os critérios de exclusão foram crianças com malformações, síndromes, deficiência auditiva e/ou visual e/ou motora, crianças inseridas em processo bilíngue de aquisição da linguagem, com déficit cognitivo que evidenciasse suspeita de deficiência intelectual importante ou com diagnóstico de TEA realizado por médico e/ou pela pontuação padrão da Escala Labirinto^(7)^.

O grupo TEA, que teve dados fornecidos pelo laboratório de pesquisa da escala, apresentou como critério de inclusão ter diagnóstico de TEA com laudo prévio e apresentar pontuação compatível com o diagnóstico pela CARS^(9)^ e Escala Labirinto^(7)^. O grupo DT, também coletado pelo mesmo laboratório, teve como critério de inclusão não apresentar atrasos, déficits ou transtornos no neurodesenvolvimento e ter todos os escores abaixo do ponto de corte para diagnóstico de TEA pela CARS^(9)^. Para minimizar o possível viés pela coleta do grupo TL ter sido realizada em época e grupo distintos, buscou-se a revisão dos casos duvidosos no diagnóstico diferencial pelo grupo Labirinto, em sessão conduzida pela Dra. Milena Pondé e pelo Dr. Gustavo Siquara, autores da escala. Nesse momento, a revisão era feita de modo que o grupo não sabia se era um caso de TL ou TEA e buscava verificar o diagnóstico diferencial. As fonoaudiólogas pesquisadoras também foram certificadas na aplicação da Escala Labirinto por ambos autores e acompanharam diagnósticos semanais durante um ano de formação.

### Instrumentos e procedimentos de coleta

Na primeira etapa foi realizado contato com as clínicas-escola de dois cursos públicos de Fonoaudiologia e as fonoaudiólogas de clínicas particulares (seis no total) para localizar os sujeitos com as características de TL ou queixa de atraso na aquisição da linguagem. Em seguida, foi realizado contato com os responsáveis pessoalmente ou via *whatsapp*, convidando-os a participarem da pesquisa. Realizou-se agendamento da avaliação presencial para os que concordaram em se voluntariar.

Além do agendamento, neste momento foi enviada a anamnese da Escala Labirinto para os responsáveis, via *Google Forms*. Foi realizado também envio do Inventário Dimensional de Avaliação do Desenvolvimento Infantil (IDADI)^(8)^, de forma *on-line* por meio da editora do teste, para os responsáveis responderem. Ressalta-se que o envio *on-line* foi feito para os participantes com acesso digital. Os que tiveram dificuldades ou limitações de acesso preencheram os protocolos na forma impressa, durante o atendimento presencial.

A etapa seguinte foi o atendimento presencial com a criança e seus responsáveis para aplicação da Escala Labirinto. O primeiro passo, com os responsáveis, foi ler presencialmente o TCLE e solicitar sua assinatura, uma vez que já haviam aceitado participar da pesquisa no contato virtual. Também nesse momento, os responsáveis que não haviam preenchido a anamnese da Escala Labirinto e o IDADI^(10)^ de forma *on-line*, preencheram-nos na forma impressa e também foi feita uma escuta dos pais para complementar informações da anamnese e saber de suas percepções sobre o filho(a).

Em seguida foi realizada aplicação da Escala Labirinto^(7)^ em seus procedimentos lúdicos de forma presencial com as crianças. A aplicação da escala ocorreu em sala clínica individual, arejada e iluminada, atendendo aos requisitos sanitários vigentes, pois ainda havia a pandemia de COVID-19 no início da coleta. As coletas foram realizadas pela pesquisadora ou pela orientadora deste estudo, ambas com certificação para realizar a avaliação. A aplicação foi realizada na presença do responsável e teve duração média de 40 a 60 minutos. Utilizaram-se os materiais necessários para aplicação a Escala Labirinto.

Na fase um: tabuleiro de encaixe de animais, dois chocalhos, torre de caixas, bola, carrinho, casa de chaves, telefone e boliche. Na fase dois: boneca, acessórios de brincadeira de cozinha – miniatura de fogão, panelas, mamadeira e comidinhas, miniaturas de escova de dentes, escova de cabelo, cama/berço. Em alguns casos foram usadas também bolhas de sabão ou balão, conforme previsto nas orientações de aplicação^(7)^.

As avaliações foram gravadas em vídeo, utilizando como dispositivo de captação de imagens o celular, apoiado em um suporte com tripé na sala. As gravações foram organizadas e armazenadas utilizando a ferramenta Google Drive. Durante a aplicação, nos primeiros cinco minutos, observou-se a exploração livre feita pela criança de alguns brinquedos deixados à disposição no ambiente. A seguir, iniciaram-se as atividades previstas para avaliação das consignas do teste.

A Escala Labirinto^(7)^ é um instrumento validado para a população brasileira, que tem como objetivo avaliar e caracterizar os sintomas centrais do TEA descritos no DSM-V^2^ em crianças de dois a quatro anos e 11 meses. A ferramenta é subdivida em Sintomas Centrais, Sintomas Associados e Alterações Somáticas Associadas. É composta por entrevista dirigida, avaliação de sintomas centrais, incluindo os domínios de Interação Social (IS), Comunicação Verbal (CV), Comunicação Não Verbal (CNV) e Comportamento Rígido e Gesto Repetitivo (CRGR), além do mapeamento de sintomas comumente associados ao TEA e alterações somáticas associadas. A partir da análise sistemática e metódica dos quatro eixos constituintes dos Sintomas Centrais, utilizando a escala *Likert,* com pontuações entre zero e cinco pontos, que caracterizam frequência e intensidade dos sintomas, define-se o ponto de coorte para diagnóstico de TEA na pontuação total e em cada subescala^(7)^.

Para análise dos sintomas centrais há a subescala Comportamento Rígido e Gesto Repetitivo (CRGR), na qual são avaliados nível de habilidade de jogo (simples, composto, simbólico, estereotipado e repetitivo), rigidez, movimentos repetitivos, movimentos de busca sensorial e interesses restritos; Comunicação Não Verbal (CNV), composta pelos itens resposta ao chamado do nome, contato visual, intenção e resposta da atenção compartilhada e gestos comunicativos; Comunicação Verbal (CV), que contempla itens de linguagem verbal expressiva, repertório linguístico a partir de parâmetros esperados para a faixa etária como uso de palavras e narrativa, uso de linguagem estereotipada e reciprocidade na comunicação que se relaciona à participação no diálogo endereçando enunciados ou respondendo ao interlocutor; Interação Social (IS), que contém os itens resposta a aproximação, busca do examinador ou familiar durante a brincadeira e sorriso social.

O primeiro procedimento do processo de avaliação é a realização da anamnese. A segunda etapa consiste na aplicação da escala, em suas atividades lúdicas. Essa parte é feita na presença da criança, do profissional e do responsável. Sugere-se que seja feita em sala individual e estima-se duração de 40 a 60 minutos para aplicação, gravada em vídeo. Os procedimentos para avaliação incluem observação do brincar espontâneo iniciado pela criança durante os primeiros cinco minutos de aplicação. Após, o avaliador conduz determinadas atividades conjuntas, em contexto naturalista de jogo, utilizando os materiais previstos no manual de aplicação para a etapa um e dois.

A Escala Labirinto também analisa sintomas associados entre os quais itens que se relacionam à Cognição, Comportamento Disruptivo, Impulsividade, Hiperatividade, Hipoatividade, Comportamento Hetero e Auto Lesivo e Comportamento Obsessivo/Compulsivo. Dentro das Alterações Somáticas estão os itens Alterações no Controle de Esfíncteres, Sono, Problemas Gastrointestinais e Respiratórios, Tônus, Síndromes, Alterações Neurológicas ou Dismorfismos, Episódios Pré ou Perinatais e Epilepsia.

Ao final da aplicação, a gravação deve ser analisada por um profissional com certificação para aplicação da Escala Labirinto. A pontuação é atribuída na ficha de respostas e são calculadas a pontuação total e por subescala. De acordo com os critérios psicométricos, o ponto de coorte para TEA deve ser igual ou maior que 12 pontos na pontuação total e igual ou maior a três na subescala IS, igual ou maior a quatro, nas subescalas de CV e CRGR, e igual ou maior a dois na subescala de CNV.

Para diagnóstico do grupo TL, além da aplicação do IDADI^(8)^ feita pelos pesquisadores e da Escala Labirinto^(7)^, foram utilizadas outras avaliações protocolares para auxiliar no diagnóstico, escolhidas pelos fonoaudiólogos que avaliaram as crianças antes da pesquisa ser realizada. Além disso, todos relataram ou utilizaram os critérios do DSM-V^2^ para identificar as manifestações clínicas dos sintomas de TL. Os profissionais utilizaram testes que acharam pertinentes para verificar a aquisição da linguagem tais como a Avaliação do Desenvolvimento da Linguagem (ADL)^(11)^ ou avaliações mais específicas de vocabulário, fluência, pragmática, fonologia (ABFW)^(10)^, praxias^(12)^.

As avaliações das 28 crianças do grupo DT e dos 48 sujeitos do grupo TEA foram realizadas em uma clínica de psicologia, mediante leitura e assinatura do TCLE pelos responsáveis. Estagiários do curso de Psicologia e médicos residentes de um curso de psiquiatria da Escola Bahiana de Medicina aplicaram os procedimentos padrões da Escala Labirinto^(7)^ e da CARS^(9)^ nas crianças de ambos grupos, supervisionados por uma psiquiatra e um psicólogo. Após, a mesma equipe pontuou as escalas e realizou diagnóstico clínico conforme critérios do DSM-V^2^.

O grupo com TL foi composto de duas formas: indicado por fonoaudiólogas de clínicas particulares e selecionados da lista de espera das clínicas-escola. Algumas crianças das clínicas particulares possuíam avaliações neuropediátricas com diagnóstico de TL, além da avaliação das fonoaudiólogas. Desse modo, buscou-se utilizar um procedimento similar ao utilizado na coleta do grupo TEA que abrangeu uma avaliação clínica de expert e um teste específico, no caso das crianças com TEA CARS e no caso das fonoaudiólogas testes de linguagem. As crianças das clínicas-escola foram selecionadas de uma lista de espera de crianças com a queixa de atraso na aquisição da linguagem e a Escala Labirinto e o IDADI foram aplicados servindo para a seleção de quem poderia ser um caso de TEA e quem seria TL. Também foram utilizados testes de linguagem e observação clínica das pesquisadoras experientes em linguagem. A cada dez crianças com essa queixa quatro eram casos de TEA e seis de TL. A Escala Labirinto apresentou-se fundamental na distinção que foi feita, nos casos duvidosos, conforme já mencionado, pelo grupo de pesquisa Labirinto.

### Análise dos dados

O material audiovisual gravado correspondente à aplicação da Escala Labirinto foi analisado pela pesquisadora deste trabalho, que atribuiu as pontuações da Escala Labirinto para cada caso avaliado. A orientadora do estudo revisou as pontuações estabelecidas. Em casos em que houve dúvida quanto aos resultados, principalmente quando a interface de TL com diagnóstico de TEA se fez presente, e nos casos de sinais clínicos evidentes de TEA, a coorientadora realizou a conferência das tabulações e cotações de pontos. Em caso de persistência da dúvida, o caso foi levado para discussão em grupo de estudo pelo laboratório de pesquisa. Não houve discordâncias superiores a 5% nas pontuações atribuídas, assim como nos diagnósticos. Para a decisão da pontuação final prevaleceu a atribuição das orientadoras deste estudo.

O IDADI^(12)^ consiste em uma avaliação abrangente do desenvolvimento infantil envolvendo sete domínios: cognitivo, socioemocional, comunicação e linguagem receptiva e expressiva, motricidade ampla e fina, e comportamento adaptativo. O inventário inclui 524 itens que descrevem comportamentos e habilidades esperadas para cada faixa etária entre os quatro e 72 meses de vida, respeitando os marcos do neurodesenvolvimento infantil. O protocolo está validado para a população brasileira e permite coletar informações do desenvolvimento infantil por meio de relatos parentais.

O questionário pode ser respondido pelos pais ou responsáveis, tanto no formato autoadministrado, quanto como entrevista a ser conduzida pelo profissional/pesquisador. A pontuação é realizada utilizando a escala *Likert*, na qual a resposta “sim” equivale a dois pontos, quando a criança domina o que está descrito no item; “às vezes”, quando executa com dificuldade e em alguns momentos, o que corresponde a um ponto; e “não”, quando a criança não executa ou nunca foi observada realizando determinada atividade, representando zero na pontuação. Ao final, é feito o escore bruto de cada domínio. Em seguida, podem ser consultados os escores padronizados e de desenvolvimento, que nesta pesquisa foi realizado no sistema *on-line* da própria editora, e enviados por e-mail para a orientadora do estudo e para os pais e fonoaudiólogos responsáveis pelos casos.

Dentre as opções de análise estão crianças que apresentam desenvolvimento muito acima do esperado, acima do esperado, típico, em alerta para atraso, com atraso ou com atraso significativo. Nesta pesquisa sua utilização buscou confirmar o diagnóstico de TL atribuído pelas fonoaudiólogas que fizeram o encaminhamento dos sujeitos para a pesquisa. Considerou-se critério para inclusão haver atraso ou atraso significativo na comunicação e linguagem expressiva e/ou compreensiva. Poderiam também haver atrasos ou alerta de atraso nas demais dimensões avaliadas desde que não se caracterizassem como casos de deficiência intelectual ou motora, entre outras, analisadas pelas especialistas. Em caso de dúvida, os casos foram descartados ou encaminhados para mais avaliações.

Após, os dados das respostas revisadas foram lançados em uma planilha excel, contendo as informações da Escala Labirinto em sua pontuação total e subescalas, considerando os três grupos estudados (DT, TEA e TL).

A análise da relação entre as variáveis coletadas foi realizada por meio do *software* JASP, versão 0.15. Foram utilizadas medidas de estatística descritiva e inferencial. Para comparar a pontuação na escala entre os grupos de crianças com DT, TL e TEA, foi utilizado o teste ANOVA *one-way*. Também foi utilizado o teste Post hoc Scheffé, que avalia o nível de confiança geral, quando os tamanhos das amostras dos grupos são diferentes, como neste estudo. Salienta-se que os materiais e códigos das análises estão disponíveis a partir de requerimento por e-mail para o autor de correspondência.

## RESULTADOS

Na Tabela 1, são apresentadas as médias e desvios padrão referentes aos grupos DT, TEA e TL na pontuação em cada subescala e na pontuação total da Escala Labirinto.

**Tabela 1:** Pontuações dos grupos na Escala Labirinto

**Grupos**	DT	TEA	TL	ANOVA
Subescalas Labirinto	N=28	N=48	N=38	p-valor
	M(DP)	M(DP)	M(DP)	Comparação três grupos
Interação social	0,5(1,2)	6,9(2,9)	1,4(1,4)	<0,001
Comunicação verbal	1,3 (2,1)	9,5(3,2)	6,8(2,1)	<0,001
Comunicação não verbal	0,7 (2,2)	13,4 (5,7)	3,3(2,4)	<0,001
Comportamentos rígidos e gestos repetitivos	0,8 (1,1)	6,5 (2,6)	2,4 (1,3)	<0,001
Pontuação total Labirinto	3,5(5,2)	37,1(12,9)	14,1 (4,9)	<0,001

**Legenda:** DT = desenvolvimento típico; TEA = transtorno do espectro autista; TL = Transtorno de linguagem; N = número; M = Média; DP = desvio padrão

Na comparação entre os grupos de crianças a partir do diagnóstico, por meio do teste ANOVA *one-way*, observa-se que há diferença significativa em relação aos grupos DT, TL e TEA referentes a cada subescala: Interação Social (p<0,001), Comunicação Verbal (p<0,001), Comunicação Não-Verbal (p<0,001), Comportamento Rígido e Gesto Repetitivo (p<0,001), Pontuação Total Escala Labirinto (p<0,001). Além disso, é possível observar a pontuação com aumento progressivo, diferenciando os grupos DT, TL e TEA em todas as subescalas e na pontuação total.

Na Figura 1 é apresentada a comparação nas pontuações de acordo com cada subescala da Escala Labirinto, para os grupos DT, TL e TEA.

**Figura 1:** Comparação no teste post hoc entre grupos DT, TL e TEA

**Legenda:** IS = Interação Social; CV = Comunicação Verbal; CNV = Comunicação Não Verbal; CRGR = Comportamento Rígido e Gesto Repetitivo. *=p<0,05. Fonte: produção da autora

Conforme análise da Figura 1, a comparação a cada dois grupos analisada com uso do teste *post hoc* utilizando a correção de Scheffé, indicou diferença significativa entre os grupos nas subescalas. Na Interação Social entre os grupos DT e TEA (p<0,001) e TL e TEA (p<0,001). No entanto, entre os grupos DT e TL não foram encontradas diferenças significativas (p=0,310).

Em relação à comparação na subescala Comunicação Verbal, encontraram-se diferenças entre os três grupos comparados (DT X TL; DT X TEA; TL X TEA) com valor de p<0,001. Já na comparação da subescala de Comunicação Não Verbal observaram-se diferenças entre os três grupos comparados (DT X TEA; TL X TEA) com valor de p<0,001 e DT X TL com valor de p=0,04). Em relação à comparação de desempenho na subescala Comportamento Rígido e Gesto Repetitivo, constataram-se diferenças entre os três grupos comparados (DT X TL; DT X TEA; TL X TEA) com valor de p<0,001.

Além disso, a análise da Figura 1 permite observar aumento crescente das pontuações em cada subescala, seguindo a ordem de grupo DT, TL e TEA. Em todas as subescalas o grupo TEA apresentou escores maiores. Apesar das pontuações de todas subescalas terem diferença estatisticamente significativa em relação ao grupo TEA, observa-se maior diferença entre o grupo TL e TEA na subescala de Comunicação Não Verbal e menor diferença na pontuação dos itens de Comunicação Verbal.

As diferenças encontradas nas subescalas avaliadas pela Escala Labirinto entre os grupos DT, TL e TEA também estão representadas na Figura 2.

**Figura 2:** Gráficos de distribuição das pontuações dos grupos DT, TL e TEA nas subescalas da Escala Labirinto

**Legenda:** IS = Interação Social; CV = Comunicação Verbal; CNV = Comunicação Não Verbal; CRGR = Comportamento Rígido e Gesto Repetitivo; DT = Desenvolvimento Típico; TL = Transtorno de Linguagem; TEA = Transtorno do Espectro Autista. Fonte: produção da autora

Na Figura 2, verifica-se que a pontuação em cada subescala da avaliação permite diferenciar claramente os grupos de crianças com DT e TL das crianças com TEA. Em relação às subescalas dos sintomas centrais que se referem a Comunicação Verbal, Comunicação Não Verbal e Comportamentos Rígidos e Gestos Repetitivos, verificou-se diferença estatística entre as crianças com DT e TL. Apenas o sintoma de Interação Social não permitiu diferenciar estatisticamente o grupo com DT e o grupo com TL.

Cabe ainda uma observação em relação às subescalas que se relaciona ao item de comportamentos rígidos e gestos repetitivos nas crianças com TL. Observou-se que as maiores pontuações se relacionaram ao subitem relativo às habilidades do brincar abaixo do esperado para a faixa etária e menos à presença de maneirismos ou gestos repetitivos.

## DISCUSSÃO

A Escala Labirinto^(7)^ é um instrumento acessível e previamente validado na realidade brasileira para caracterizar e auxiliar o clínico a realizar diagnóstico de TEA em crianças de dois a quatro anos e 11 meses na população brasileira. Os resultados deste estudo indicam que a escala pode auxiliar no diagnóstico diferencial entre as crianças com TEA e TL, considerando-se que os escores de todas as subescalas que avaliam os Sintomas Centrais apresentaram diferenças estatisticamente significativas no grupo de crianças TL e TEA. As subescalas de Interação Social, Comunicação Não Verbal, Comunicação Verbal e Comportamento Rígido e Gesto Repetitivo também diferenciaram as crianças em DT de TL em três das quatro dimensões avaliadas. Os grupos TL e TEA apresentaram os mesmos sintomas clínicos, no entanto, observam-se pontuações maiores no segundo grupo em relação ao primeiro. Deste modo, a Escala Labirinto pode ser um instrumento que discrimine casos em que podem emergir dúvidas como ressaltado por Markiewics e Pachalska^(6)^, quando ressaltam as dificuldades inerentes ao diagnóstico diferencial entre TEA e TL em crianças abaixo de três anos, pela interface comum das alterações de linguagem em ambos casos.

A queixa inicial de atraso na linguagem é comumente associada à ausência ou presença de fala, aspecto que tomou proporções preocupantes no período da pandemia da COVID 19^(13)^, momento no qual esta pesquisa foi realizada. Esse fator fez os responsáveis buscarem atendimento, tanto para os casos de TEA, quanto para os casos de TL. É importante destacar que, apesar de os pais perceberem mais as alterações de fala, os déficits comunicativos não estão presentes apenas no âmbito da linguagem oral, mas também na gestual. Isso é claramente demonstrado na análise das Figuras 1 e 2. Nos gráficos de Comunicação Verbal e Não Verbal da Figura 2 isso é muito evidente. Não apenas na subescala de Comunicação Verbal os grupos TEA e TL apresentaram déficits. Na subescala de Comunicação Não Verbal os atrasos são evidentes, em ambos grupos, mas de modo mais importante no grupo com TEA. Isso demonstra como a interface entre comunicação oral e gestual torna-se substancial quando se assume um conceito de linguagem na perspectiva multimodal. Nesta, constrói-se uma matriz de funcionamento linguístico cognitivo na qual o gesto se apresenta integrado a produção vocal^(14)^.

Por isso, quando há atraso evidente em comunicação verbal, é necessário observar as habilidades de comunicação não verbal também. Neste estudo, a Escala Labirinto^(7)^ mostrou-se capaz de identificar atraso em ambas as subescalas, sendo que no TEA as pontuações são consideravelmente mais elevadas em relação ao grupo TL.

Esses fatores já foram evidenciados em outros estudos, que apontam que no TEA há padrões atípicos no uso de gestos com função comunicativa. Além de produzir menos gestos, as crianças com TEA têm déficits principalmente em realizar gestos que envolvem habilidades de atenção compartilhada, e em integrar olhar e gesto para se comunicar, sobretudo a partir dos 12-13 meses de idade^(15)^. Esses achados evidenciam a importância de se considerar a pontuação da comunicação não verbal no diagnóstico diferencial de TEA, TL e DT.

Na subescala de Interação Social, o grupo TL não apresentou diferença significativa do grupo DT, conforme representam a Tabela e Figura 1. Este aspecto pode indicar que crianças com TL não possuam problemas nas interações sociais dadas suas condições intelectuais e socioemocionais, como prevê a literatura clássica que chegou a chamar esses distúrbios de específicos à linguagem^(14)^.

Nesta mesma subescala, entretanto, no que tange à distinção entre o grupo TEA e TL, as diferenças nos escores são claras. O grupo com TEA pontuou significativamente acima do grupo com TL. Cabe citar o estudo de Simms e Jin^(15)^, que analisou as diferenças entre os sintomas de crianças com TEA e TL, conforme DSM^(2)^, e indicou que crianças com TL têm maior interesse social, habilidades de atenção compartilhada mais consolidadas e mais reciprocidade afetiva. O mesmo estudo salientou que déficits no âmbito da interação social são esperados, mas como consequência das dificuldades linguísticas básicas.

Na última subescala analisada, conforme Figuras 1 e 2, a subescala de Comportamento Rígido e Gesto Repetitivo, repetiu-se o padrão analisado nas demais subescalas. Grupo TEA com maior pontuação estatisticamente significativa em relação ao grupo TL e DT. Esse resultado está em conformidade com os comportamentos já citados na literatura quando se trata da sintomatologia do TEA. As crianças com TEA manifestam interesses mais restritos e hiperfoco em determinados tópicos ou objetos que limitam seu interesse nos demais, inflexibilidade e rigidez com rotinas, manuseios atípicos de objetos, além de estereotipias vocais ou motoras^(2,16)^.

Apesar de obterem menor pontuação na subescala de Comportamento Rígido e Gesto Repetitivo do que o grupo TEA, é interessante analisar que os casos TL pontuam significativamente mais alto do que os casos DT. Dentre os quatro itens que compõem este domínio, o grupo TL teve escores mais altos principalmente em movimentos repetitivos/estereotipados e brincar/simbolização.

Neste âmbito, casos de estereotipias motoras em crianças com TL, comumente um sinal de alerta para TEA, já foram apontados em estudo que identificou estereotipias motoras semelhantes às manifestações características do TEA, em dois casos de crianças com TL. Em outro estudo, que analisou 277 crianças com diferentes diagnósticos, incluindo TEA, foi constatado que 18,3% das crianças com TL apresentaram algum tipo de estereotipia^(17)^.

Outro fator que contribuiu para o aumento na pontuação do grupo TL, diferenciando-o do grupo DT na subescala de Comportamento Rígido e Gesto Repetitivo, foi o item de habilidades de brincadeira. Observa-se que tanto a linguagem como o brincar simbólico exige capacidade de representação, abstração e imaginação^(18,19).^ Crianças que atingem níveis mais complexos de jogo também apresentam melhor desempenho em avaliações cognitivas e comunicativas^(18,19)^.

Nas crianças com TL, observam-se habilidades de jogo mais pobres. O perfil de características encontradas em crianças com TL aponta para ocorrência de menos variações de brincadeira, menos iniciações de rotinas, menor repertório de jogo e principalmente, menos condutas simbólicas no nível de faz-de-conta^(19-23)^. Isso se confirmou neste estudo já que, apesar do grupo TEA apresentar as maiores pontuações nesse item da subescala, demonstrando um padrão de jogo mais restrito, com menos condutas funcionais, o grupo TL, em menor escala, também apresentou dificuldades em demonstrar habilidades de brincadeira criativas e flexíveis, principalmente em níveis simbólicos, como atribuir vida a um boneco, usar um objeto como se fosse outra coisa e imaginar a presença de um objeto sem a presença concreta do item^(18)^.

Considera-se que entre os pontos fortes da utilização da Escala Labirinto estão a formação conjunta da equipe multidisciplinar, a concepção abrangente de desenvolvimento que a embasa, pois há parâmetros evolutivos que servem de referência e criam uma linha de base para a observação realizada a partir da filmagem das tarefas propostas, minimizando um olhar subjetivo. Outro ponto forte é ser acessível financeiramente a todos os profissionais clínicos que a utilizam, quer pelo acesso à formação *on-line* que pode ser feita de qualquer parte do Brasil ou pela aquisição acessível dos brinquedos e materiais necessários a sua aplicação. Esses pontos fortes se refletiram nesta pesquisa. Além disso, o fato de que as coletas se do grupo TL se deram em cidades distintas do estado e com casos atendidos em clínicas-escola e particulares, é outro ponto forte, pela diversidade de situações econômicas e culturais dos casos analisados.

Em relação aos pontos fracos da pesquisa, destacamos o fato de ter sido possível uma coleta conjunta e cega de ambos os grupos, com TEA e TL. Como o estudo com TL foi feito logo após a validação da escala, acredita-se que estudos futuros poderão minimizar esse ponto fraco.

## CONCLUSÃO

A escala Labirinto apresentou-se efetiva na diferenciação entre o grupo com TEA e o grupo com TL e entre as crianças com TEA e com DT. Todas as subescalas também diferenciaram os três grupos, a exceção da escala de interação social não evidenciou distinção significativa entre os grupos com TL e DT.

A pesquisa sugere que para uma comprovação mais efetiva, ela deve ser investigada com amostras maiores e em situações clínicas em que o diagnóstico é desconhecido, para verificar sua eficácia no diagnóstico diferencial.
